# Neurosurgical Management of Myelomeningocele in Premature Infants: A Case Series

**DOI:** 10.21203/rs.3.rs-4158288/v1

**Published:** 2024-04-02

**Authors:** Addison Stewart, Andrew T. Hale, Benjamin W. Saccomano, Ariana S. Barkley, Betsy D. Hopson, Anastasia Arynchyna-Smith, James M. Johnston, Brandon G. Rocque, Jeffrey P. Blount, Curtis J. Rozzelle

**Affiliations:** Children’s of Alabama; Children’s of Alabama; Children’s of Alabama; Children’s of Alabama; Children’s of Alabama; Children’s of Alabama; Children’s of Alabama; Children’s of Alabama; Children’s of Alabama; Children’s of Alabama

## Abstract

**Introduction:**

Myelomeningocele (MMC) is the most common neural tube defect, but rarely seen in premature infants. Most centers advocate for closure of MMC within 24 hours of birth. However, this is not always possible in severely premature infants. Given the rarity of this patient population, we aimed to share our institutional experience and outcomes of severely premature infants with MMC.

**Methods:**

We performed a retrospective, observational review of premature infants (≤ 32 weeks gestational age) identified through our multidisciplinary spina bifida clinic (1995–2021) and surgical logs. Descriptive statistics were compiled about this sample including timing of MMC closure and incidence of adverse events such as sepsis, CSF diversion, meningitis, and death.

**Results:**

Eight patients were identified (50% male) with MMC who were born ≤ 32 weeks gestational age. Mean gestational age of the population was 27.3 weeks (SD 3.5). Median time to MMC closure was 1.5 days (IQR = 1 −80.8). Five patients were taken for surgery within the recommended 48 hours of birth; 2 patients underwent significantly delayed closure (107 and 139 days); and one patient’s defect epithelized without surgical intervention. Six of eight patients required permanent cerebrospinal fluid (CSF) diversion (2 patients were treated with ventriculoperitoneal shunting (VPS), three were treated with endoscopic third ventriculostomy (ETV) with choroid plexus cauterization (CPC) and 1 patient treated with ETV; mean of 3 years after birth, ranging from 1 day to 16 years). Two patients required more than one permanent CSF diversion procedure. Two patients developed sepsis (defined as meeting at least 2/4 SIRS criteria), and 2 patients had intraventricular hemorrhage (both grade III). No patients developed meningitis (defined as positive CSF cultures) prior to MMC closure. Median follow up duration was 9.7 years. During this time epoch, 3 patients died: Two before 2 years of age of causes unrelated to surgical intervention. One of the two patients with grade III IVH died within 24 hours of MMC closure.

**Conclusions:**

In our institutional experience with premature infants with MMC, some patients underwent delayed MMC closure. The overall rate of meningitis, sepsis, and mortality for preterm children with MMC was similar to MMC patients born at term.

## Introduction

Myelomeningocele (MMC) is the most severe form of spina bifida compatible with life and the most common congenital abnormality of the central nervous system [1, 2]. Failure of neural tube closure often results in multiple clinical sequelae including hydrocephalus, loss of motor and sensory function, bladder and bowel dysfunction and musculo-skeletal deformities [3]. In the United States, approximately 1,400 children are born with a neural tube defect every year [4]. Historically, closure within 48 hours has been recommended to reduce the risk of infection [5]. Observational data have shown that aggressive early surgical treatment could decrease the incidence of complications and overall morbidity of the condition [5].

In some circumstances (e.g., severe prematurity) MMC closure is not feasible within the 48-hour window recommended by the Congress of Neurological Surgeons Guidelines for Pediatric Myelomeningocele [6, 7]. Pre-term infants have increased risk of sepsis, intraventricular hemorrhage (IVH), meningitis, necrotizing enterocolitis, and low birth weight, all of which present challenges for immediate surgical intervention [8]. Often, surgery must be delayed in pre-term children to mitigate the numerous complications of prematurity. However, to our knowledge, there are no studies documenting the care and management of pre-term infants with MMC. This study serves to characterize a rare cohort of patients who were pre-term with a MMC, with no history of attempted fetal surgery.

## Methods

We performed a retrospective review of premature (born at ≤ 32 weeks gestational age) infants born between September 2001 and June 2021 with MMC who were treated at Children’s of Alabama. Institutional Review Board approval (IRB-300003587) was obtained for this retrospective observational study. Patients were analyzed from birth to date of last follow-up. The primary outcome variables of this study were time to MMC closure, incidence of intraventricular hemorrhage (IVH), and incidence of sepsis. IVH was assessed using standard ultrasound techniques. Grading of IVH was determined in accordance with the Papile grading system severity scale, as noted in the ultrasound report [9, 10]. Incidence of sepsis was assessed based on analysis of ICD-10 code A41.9 for sepsis of unspecified organism. This code is assigned to all patients at our center who meet 2 or more Systemic Inflammatory Response (SIRS) criteria [11]. Additionally, we recorded both clinical and demographic variables including ethnicity, sex, and head circumference at birth. Descriptive statistics for categorical and continuous variables were performed.

## Results

Thirteen patients were identified who were born at or before 32 weeks’ gestation and diagnosed with MMC. Of the thirteen patients, one underwent prenatal MMC closure at an outside hospital and was excluded from analysis. Another 4 patients, although managed at COA, were born at outside institutions, lacked complete birth data, and were consequently excluded from analysis. Thus, our final cohort contained eight patients who were born at or before 32 weeks’ gestation and diagnosed with MMC. Of the eight patients, five patients had MMC closure performed within the recommended window of 48 hours post birth; two experienced delayed closure (107 and 139 days), and one patient’s defect epithelized without surgical intervention. Mean gestational age at birth was 27.3 weeks (SD 3.5) with the earliest birth occurring at 23 weeks’ gestation. Median time to MMC closure was 1.5 days (IQR 1 −80.8).

Six patients (75%) required permanent cerebrospinal fluid (CSF) diversion, defined as undergoing shunt placement, ETV + CPC, or ETV alone (Table 1). Two patients were treated with ventriculoperitoneal shunting; three were treated with endoscopic third ventriculostomy (ETV) with choroid plexus cauterization (CPC); and 1 patient treated with ETV alone. Timing of permanent CSF diversion occurred at a median of 95 days (IQR 50.8–134.0), with the earliest diversion occurring at 1 day old and the latest occurring at 16 years of age. Three patients required VSGS placement during their management. Of the three patients with VSGS placement, all of the patients required future permanent diversion procedure. There were no consistent complications in VSGS placement, although one of the three patients required VSGS revision before his permanent CSF diversion procedure. This data is further summarized in Table 2.

Two patients developed sepsis (2/4 SIRS criteria), and none developed meningitis (positive CSF cultures before MMC closure). Two patients experienced intraventricular hemorrhages, both grade III. One patient, born at 23 weeks’ gestation, experienced grade III IVH shortly after birth, before any neurosurgical intervention. The other, born at 29 weeks’ gestation, experienced Grade III IVH within 24 hours of MMC closure. This patient died shortly thereafter.

Median follow up duration was 1.1 years (4 months - 16 years). 3 patients died. One 14-month-old patient died due to complications of a viral upper respiratory infection. One 6-month-old patient died of renal failure. One patient died within 24 hours of closure due to severe IVH. Individual patient characteristics are presented in Table 2.

## Discussion

Children born both pre-term and with a diagnosis of MMC are rare outside the setting of prenatal surgical MMC treatment. To our knowledge, there are no published reports of this unique cohort. For patients with MMC, aggressive early treatment of neural tube defects decreases morbidity of the disease and decreases the rate and severity of the associated neurological and physical deficits [12]. The widely accepted and traditional recommendation is to close all MMC deficits within 48 hours of birth [6, 7], largely because of the theoretical risk of sepsis and meningitis. Yet, due to the complications of prematurity, MMC defect closure may be delayed beyond the recommended window for closure.

In our cohort of premature infants with MMC, the majority underwent MMC closure within the 48-hour after birth recommended window. Only the most premature 3 patients, all born under 25 weeks, were managed with delayed closure. In these 3 patients there was no significant difference in overall outcome, incidence of IVH, sepsis, and CSF diversion procedures. We observed a single instance of fatal IVH within 24 hours of operation in a premature infant, born at 29 weeks, who underwent MMC within 48 hours of birth. This particular instance was early in the series and served as the catalyst for recommending delayed closure in this population at our institution. In other premature patients with delayed closure of their MMC defect, no such hemorrhage was observed. This observation suggests that delayed closure may not be detrimental in premature infants.

Approximately 80% of patients with MMC will develop hydrocephalus necessitating permanent CSF diversion [13, 14]. In our cohort, 6/8 (75%) of patients required permanent CSF diversion. Our study suggests that rates of permanent CSF diversion in pre-term infants with MMC are comparable to those with MMC born at term. Furthermore, rates of meningitis, sepsis, and mortality for MMC closure with 48 hours of birth have been reported at 16–19%, 3%, and 0% respectively [15, 16, 17]. In our cohort these outcomes occurred at similar rates; 0%, 9%, and 9%. In pediatric patients, the rate of shunt revision has been reportedly high [12]. In a large, multi-center study, the occurrence of shunt failure during 3 years of follow up was 33% [18]. In our cohort, only 2/8 (25%) of patients required shunt revision during the time of follow-up. While the average age of last follow up in our study was 8 years old, our findings suggest that rates of shunt revisions may be comparable in this population. However, 3/8 (37.5%) of patients died during routine follow-up and management in our cohort.

## Limitations

This study was performed at a single institution, and thus lacks generalizability. Nevertheless, this center services 95% of Alabama’s pediatric spina bifida population thereby having limited referral bias. No standard metrics nor treatment paradigms were applied. Clinical decisions were made by surgeon preference to optimize projected clinical outcome. During this time interval 6 attending surgeons provided care for these infants, so there is potential for bias introduced by practice preference. However, our practice embraces standard principles regarding key clinical decisions such as treatment thresholds for hydrocephalus, candidacy for shunt/ETV and closure techniques. Additionally, as medical treatment evolves there has been a shift to use of ETV-CPC in the last decade, with lower overall shunt rate. Even with this evolution, our practice strives to keep principles of management consistent. This study was conducted as a small, retrospective analysis, pre-disposing this study to single-group threat, historical threat, and maturation threat.

## Conclusions

While some patients underwent delayed closure, the overall rate of meningitis, sepsis, and mortality for preterm children with MMC was like those born at term. Nearly all patients required permanent CSF diversion. Overall, this limited cohort suggested that outcomes for premature infants with MMC were like term infants with MMC, without significant consequences for delayed closure. However, premature infants with MMC overall, may have poorer outcomes as evidenced by the increased all-cause mortality rate during the follow-up period. Further investigation into the management of pre-mature children with MMC is warranted and necessary.

## Figures and Tables

**Table 1:** Baseline patient characteristics.

**Race**	
African American	5.0 (63%)
Caucasian	2 (25%)
Hispanic/Latino	1.0 (13%)
**Head Circumference (z-score)**	
Average	−1.1
Median	−0.7
Mode	NA
Min	−3.5
Max	1.2
**IVH On First Head US**	
Yes	2.0 (25%)
Unknown	2.0 (25%)
**VSGS Placed**	
Yes	3.0 (38%)
**Permanent CSF Diversion**	
VP Shunt	2.0 (25%)
ETV/CPC	3.0 (38%)
ETV	1.0 (12%)
None	1.0 (12%)
**Time To Permanent CSF Diversion (Days)**	
Average	1074.3
Max	6072
Min	1
Standard Deviation	2448.9
**Did The Patient Require Additional Permanent Diversion Procedure?**	
Yes	2.0 (16%)
**Time To MMC Closure (Days)**	
Average	35.9
Max	139
Min	0
Unknown	4
Standard Deviation	60.2
**Corrected Age at MMC Closure (Weeks)**	
Average	−3.8
Max	2.9
Min	−11.9
Standard Deviation	5.6
**Gestational Age (Weeks)**	
Average	27.3
Median	28
Mode	28
Max	32
**Sepsis At Birth**	
Yes	1.0 (12%)
**Late Onset Sepsis**	
Yes	1.0 (12%)

**Table 2:** Individual patient characteristics.

Identifier	GA (weeks)	MMC Closure w/in 48 hrs	Permanent CSF Diversion	VSGS	Sepsis	IVH	Deceased
1	23	o	x	x	o	x	o
2	24	o	x	o	o	o	o
3	28	x	x	o	o	o	o
4	28	x	x	x	o	o	
5	31	x	x	x	o	o	o
6	32	x	x	o	x	o	x
7	23	Epi	o	o	x	o	o
8	29	x	o	o	o	x	x

GA = Gestational Age, CSF = Cerebrospinal fluid, MMC = Myelomeningocele, IVH = Intraventricular Hemorrhage, epi = epithelialized, x = positive for variable, o = negative for variable, N/A = not available, Hrs = hours
